# From Aromatic Motifs to Cluster-Assembled Materials: Silicon–Lithium Nanoclusters for Hydrogen Storage Applications

**DOI:** 10.3390/molecules30102163

**Published:** 2025-05-14

**Authors:** Williams García-Argote, Erika Medel, Diego Inostroza, Alejandro Vásquez-Espinal, José Solar-Encinas, Luis Leyva-Parra, Lina María Ruiz, Osvaldo Yañez, William Tiznado

**Affiliations:** 1 Centro de Investigación para el Diseño de Materiales (CEDEM), Facultad de Ciencias Exactas, Departamento de Ciencias Químicas, Universidad Andrés Bello, Avenida República 275, Santiago 8370146, Chile; w.garcaargote@uandresbello.edu; 2 Departamento de Química, División de Ciencias Básicas e Ingeniería, Universidad Autónoma Metropolitana, Iztapalapa CP 09340 CDMX, Mexico; erikamedel@live.com.mx; 3 Departamento de Física, Facultad de Ciencias, Universidad de Chile, Ñuñoa, Santiago 7800024, Chile; dinostro92@gmail.com; 4 Química y Farmacia, Facultad de Ciencias de la Salud, Universidad Arturo Prat, Casilla 121, Iquique 1100000, Chile; alvasquez@unap.cl; 5 Laboratory of Theoretical Chemistry, Faculty of Chemistry and Biology, University of Santiago de Chile (USACH), Santiago 8370146, Chile; jose.solar@usach.cl; 6 Centro de Investigación en Ingeniería de Materiales (CIIM), Facultad de Ingeniería y Arquitectura, Universidad Central de Chile (UCEN), Santa Isabel 1186, Santiago 8370146, Chile; luis.leyva@ucentral.cl; 7 Institute of Biomedical Sciences, Faculty of Health Sciences, Universidad Autónoma de Chile, Santiago 8910060, Chile; 8 Centro de Modelación Ambiental y Dinámica de Sistemas (CEMADIS), Facultad de Ingeniería y Negocios, Universidad de Las Américas, Santiago 7500975, Chile; oyanez@udla.cl

**Keywords:** hydrogen storage materials, silicon–lithium clusters, density functional theory, molecular dynamics, adsorption energy

## Abstract

Silicon–lithium clusters are promising candidates for hydrogen storage due to their lightweight composition, high gravimetric capacities, and favorable non-covalent binding characteristics. In this study, we employ density functional theory (DFT), global optimization (AUTOMATON and Kick–MEP), and Born–Oppenheimer molecular dynamics (BOMD) simulations to evaluate the structural stability and hydrogen storage performance of key Li–Si systems. The exploration of their potential energy surface (PES) reveals that the true global minima of Li_6_Si_6_ and Li_10_Si_10_ differ markedly from those of the earlier Si–Li structures proposed as structural analogs of aromatic hydrocarbons such as benzene and naphthalene. Instead, these clusters adopt compact geometries composed of one or two Si_4_ (*T_d_*) units and a Si_2_ dimer, all stabilized by surrounding Li atoms. Motivated by the recurrence of the Si_4_–*T_d_* motif, we explore oligomers of Li_4_Si_4_, which can be viewed as electronically transmuted analogues of P_4_, confirming the additive H_2_ uptake across dimer, trimer, and tetramer assemblies. Within the series of Si–Li clusters evaluated, the Li_12_Si_5_ sandwich complex, featuring a σ-aromatic Si_5_^10−^ ring encapsulated by two Li_6_^5+^ moieties, achieves the highest hydrogen capacity, adsorbing 34 H_2_ molecules with a gravimetric density of 23.45 wt%. Its enhanced performance arises from the high density of accessible Li^+^ adsorption sites and the electronic stabilization afforded by delocalized σ-bonding. BOMD simulations at 300 and 400 K confirm their dynamic stability and reversible storage behavior, while analysis of the interaction regions confirms that hydrogen adsorption proceeds via weak, dispersion-driven physisorption. These findings clarify the structure–property relationships in Si–Li clusters and provide a basis for designing modular, lightweight, and thermally stable hydrogen storage materials.

## 1. Introduction

Hydrogen is widely regarded as a promising energy carrier due to its high gravimetric energy density, environmental compatibility, and potential role in decarbonizing multiple sectors of the global energy system. However, its practical implementation is limited by the lack of storage technologies that simultaneously ensure safety, reversibility, and efficiency under ambient or near-ambient conditions [1,2,3]. Conventional storage methods such as high-pressure compression and cryogenic liquefaction suffer from energy inefficiency, safety risks, and low volumetric densities. In contrast, material-based hydrogen storage—where hydrogen is stored via physisorption, chemisorption, or a combination of both—has emerged as a compelling alternative [4,5]. For such systems to be practical, the adsorption energies must typically fall within the range of −0.1 to −0.8 eV per H_2_ molecule, striking a balance between a sufficient binding strength and reversible release under operational conditions [6,7,8]. Recent reviews have comprehensively surveyed hydrogen storage technologies, including solid-state systems, porous frameworks (e.g., MOFs, COFs), and nanostructured materials, emphasizing advances in the adsorption mechanisms, gravimetric storage targets, and broader system-level challenges [9,10,11,12]. Theoretical studies have shown that nanostructured materials—including metal–organic frameworks, functionalized carbon materials, and transition-metal-decorated systems—can meet these criteria through fine-tuning of their surface electronic structure, pore architecture, and active site polarity [13,14,15]. Within this landscape, atomically precise clusters offer several intrinsic advantages, including high surface-to-volume ratios, tunable reactivity, and well-defined sorption sites, positioning them as promising candidates for high-performance hydrogen storage applications.

Various silicon–lithium (Si–Li) clusters have been proposed for hydrogen storage, supported by computational predictions of high gravimetric capacities and adsorption energies compatible with reversible, non-dissociative adsorption [16,17,18,19,20]. Particular attention has been paid to aromatic clusters, combining enhanced stability with favorable interaction profiles. Si_5_Li_6_ (*C_2v_*), as studied by Jena et al. in 2012, was predicted to adsorb up to 14 H_2_ molecules, though steric constraints limit its effective uptake at around 10, with adsorption energies in the 0.11–0.16 eV per H_2_ range [16]. Shortly thereafter, in 2012, Pan, Merino, and Chattaraj reported Si_5_Li_7_^+^ (*D_5h_*) and Si_4_Li_4_ (*T_d_*) as viable hydrogen hosts, capable of storing 10–12 H_2_ molecules with gravimetric capacities of 15.25 wt% and 14.7 wt% and adsorption energies in the 0.10–0.20 eV per H_2_ range [18]. Guo and Wang, in 2020, investigated SiLi_4_^+^, composed of a central Si atom tetrahedrally coordinated by Li atoms, which adsorbs 12 H_2_ molecules at 30.2 wt% and ~0.12 eV per H_2_, with dynamic stability confirmed at 300 K via molecular dynamics simulations [19]. All of these clusters—Si_4_Li_4_, Si_5_Li_6_, Si_5_Li_7_^+^, and SiLi_4_^+^—were confirmed as global minima (GMs) through exploration of their systematic potential energy surfaces (PESs), reinforcing the reliability of their predicted properties [19,21,22,23]. In contrast, larger clusters such as Si_6_Li_6_ (*D_2h_*) and Si_10_Li_10_ (*C_s_*), proposed by Jaiswal and Sahu in 2022 [17], were constructed without global optimization. While they were predicted to adsorb 18 and 40 H_2_ molecules, respectively, with gravimetric capacities of 14.7 wt% and 18.7% and adsorption energies ranging from 0.059 to 0.141 eV per H_2_, their thermodynamic relevance remains uncertain. Larger silicon-based assemblies have also been proposed, including Li-functionalized Si_20_H_20_ frameworks with lithium-containing organic groups (e.g., CN_2_HLi, CONHLi), reported in 2015 to adsorb up to 60 H_2_ molecules (12.5 wt%) [24], and Li_12_Si_60_H_60_, a 2009 silicon analog of a decorated fullerene predicted to bind 30 H_2_ molecules (7.46 wt%) [20]. However, these extended systems have not undergone global optimization, leaving their stability and practical viability unverified.

Cluster-assembled materials (CAMs) [25], constructed from discrete, atomically defined units that retain their structural and electronic identity upon aggregation, provide a robust framework for the modular design of functional nanomaterials [26]. In the realm of Si–Li systems, our computational studies have shown that Li_4_Si_4_ (*T_d_*) [27] and Li_6_Si_5_ (*C_2v_*) [28,29]—both global minima—are promising building blocks stabilized by distinct forms of aromaticity. Li_4_Si_4_ exhibits spherical σ-aromaticity, while Li_6_Si_5_ features both σ- and π-aromatic delocalization. These clusters assemble into stable oligomers such as Li_8_Si_8_, Li_10_Si_9_, and Li_12_Si_10_, which preserve their local bonding environments and remain dynamically stable even at elevated temperatures [21]. Notably, these systems’ Si_4_^4−^ and Si_5_⁶^−^ motifs are also present in experimental Zintl phases such as Li_12_Si_7_, Li_8_MgSi_6_, and Li_21_Si_5_, which have been characterized using techniques such as X-ray diffraction and solid-state NMR spectroscopy [30,31,32,33]. In addition, lithium–silicon alloys with similar compositions have been experimentally shown to reversibly store hydrogen, reaching capacities up to 5.4 wt% under moderate conditions [34]. These precedents support the chemical plausibility and relevance to hydrogen storage of the nanoclusters explored in this work. Among these assemblies, (Li_4_Si_4_)_n_ oligomers are particularly attractive for hydrogen storage due to their high density of surface-accessible Li^+^ centers, which offer multiple binding sites for physisorption.

This study examines a series of structurally diverse Si–Li clusters selected for their potential to enable hydrogen storage via polar Li^+^ adsorption centers, favorable charge distribution, and electronically stable, modular architectures. Although distinct in topology, all analyzed systems share a common underlying motivation: they are either based on aromatic Si–Li motifs or serve as computational models of CAMs with accessible surfaces for physisorption. Firstly, we revisit the Si_6_Li_6_ and Si_10_Li_10_ clusters, previously proposed as high-capacity sorbents due to their resemblance to benzene and naphthalene, respectively [17]. While these structures suggest delocalized bonding frameworks, they have been modeled without global optimization, and their thermodynamic viability remains unresolved. Therefore, we comprehensively explore their potential energy surfaces (PESs) to locate the true global minima and reassess their hydrogen adsorption properties. In addition, we analyze the Li_12_Si_5_ (*D_5_h*) cluster [22] recently reported by our group as a global minimum sandwich-type system built from a Si_5_^10−^ ring flanked by two Li_6_^5+^ units [22]. This compact, highly polarizable structure features σ-aromatic delocalization and a high density of Li^+^ sites favorable for hydrogen binding. Finally, we investigate (Li_4_Si_4_)_n_ oligomers (n = 1–3), which model CAMs based on the Li_4_Si_4_ (*T_d_*) global minimum and exhibit a large number of accessible Li^+^ adsorption sites. Together, these systems span a range of structural motifs and degrees of modularity, allowing us to examine the interplay between PES stability, electronic structure, and hydrogen uptake capacity in Si–Li nanocluster design.

## 2. Results and Discussion

### 2.1. Confirming the Lowest-Energy Li-Si Structures

**Figure 1:**
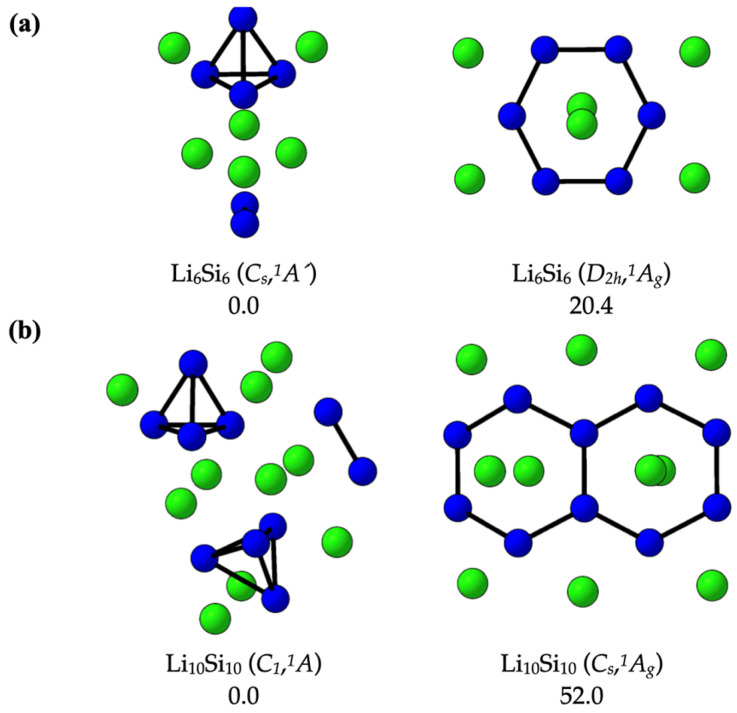
Global minima and previously proposed high-symmetry structures for (**a**) Li_6_Si_6_ and (**b**) Li_10_Si_10_ clusters. Relative energies are given in kcal·mol^−1^, computed at the DLPNO-CCSD(T)/CBS//PBE0-D3/def2-TZVP level of theory.

Figure 1 shows the global minima and the previously proposed high-symmetry structures for the Li_6_Si_6_ and Li_10_Si_10_ clusters [17], as identified through our comprehensive PES exploration. For Li_6_Si_6_ (panel a, left), the global minimum exhibits *C_s_* symmetry (*^1^A′ state*) and is composed of a Si_4_ tetrahedral unit (*T_d_*) and a Si_2_ dimer, surrounded by asymmetrically distributed Li atoms. This compact three-dimensional configuration is significantly more stable—by 20.4 kcal·mol^−1^—than the previously proposed *D_2h_*-symmetric structure (panel a, right), which features a planar Si_6_ ring and was modeled as a benzene analog in earlier hydrogen-storage-related studies. For Li_10_Si_10_ (panel b), the global minimum adopts *C_1_* symmetry (*^1^A′ state*) and consists of two Si_4_ tetrahedra and one Si–Si dimer, coordinated by a spatially dispersed set of lithium atoms. This low-symmetry arrangement lies 52.0 kcal·mol^−1^ below the *C_s_*-symmetric isomer (panel b, right), previously proposed as a naphthalene-like candidate for molecular hydrogen adsorption. These results indicate that the earlier high-symmetry, π-aromatic-like structures do not correspond to thermodynamically favored forms, as they lie significantly higher in energy than the true global minima. This highlights the necessity of a comprehensive PES exploration in cluster systems, where symmetry-based models may miss more stable, low-symmetry configurations. Notably, the global minima identified here feature Si_4_-based building units, whose recurrence points to their stabilizing role in lithium–silicon chemistry and their relevance to the design of hydrogen storage clusters. The structural diversity near the global minima is further illustrated by additional low-energy isomers within 20 kcal·mol^−1^, as reported in Figures S1 and S3 (Supporting Information).

**Figure 2:**
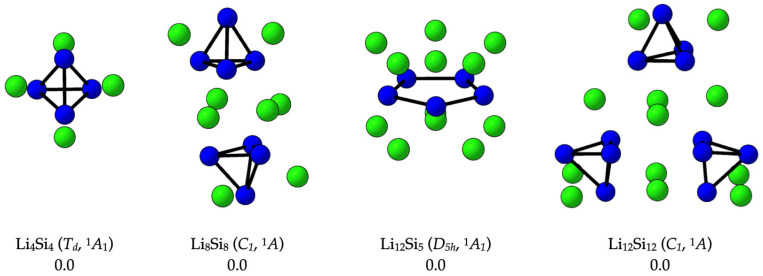
Optimized structures of global minima for the clusters Li_4_Si_4_, Li_8_Si_8_, Li_12_Si_5_, and Li_12_Si_12_, as confirmed in this work through potential energy surface (PES) exploration. All relative energies (in kcal·mol^−1^) were computed at the DLPNO-CCSD(T)/CBS//PBE0-D3/def2-TZVP level of theory.

In parallel with our identification of new global minima, we re-evaluated the potential energy surfaces of two previously proposed systems—Li_12_Si_5_ (*D_5_h*, ^1^A_1_) and the (Li_4_Si_4_)_n_ oligomers (n = 2–3)—reported initially as global minima and selected here due to their relevance to hydrogen storage. Our independent PES analysis confirms that these structures correspond to ground-state configurations. As shown in Figure 2, the Li_4_Si_4_ (*T_d_*) monomer maintains its structural integrity upon oligomerization, with Li_8_Si_8_ and Li_12_Si_12_ preserving the local Si_4_-based connectivity and Li^+^ coordination. The Li_12_Si_5_ cluster was likewise verified to occupy the lowest point on the potential energy surface. These results substantiate the thermodynamic stability of the selected clusters and support their role as structurally persistent, modular building units for hydrogen-rich Si–Li assemblies.

### 2.2. The Structural and Electronic Features of Bare and Hydrogen-Adsorbed Si–Li Clusters

Table 1 depicts the interatomic distances computed for the Si–Li clusters examined in this study and their corresponding hydrogen-adsorbed complexes. The Si–Si bond lengths remain largely invariant upon H_2_ adsorption, spanning 2.12–2.57 Å, reflecting the structural integrity of the silicon frameworks. The Si–Li distances in the bare clusters range from approximately 2.40 to 2.76 Å and undergo modest elongation upon hydrogenation, particularly in larger assemblies such as Li_8_Si_8_ and Li_12_Si_5_, where the maximum distances approach ~2.89 Å. The Li–H separations offer insight into adsorption, with the shortest distances (2.08–2.30 Å) corresponding to favorable electrostatic interactions with the exposed Li^+^ centers. As the hydrogen coverage increases, longer Li–H contacts—up to ~3.9 Å—are observed, indicating weaker physisorption at more peripheral or sterically hindered sites. This trend appears in both the global minimum (GM) structures identified through PES exploration and in the previously proposed high-symmetry local minima, Li_6_Si_6_* and Li_10_Si_10_*. While both structures support molecular hydrogen adsorption, the GM isomers generally present more spatially accessible and topologically diverse Li^+^ coordination, which can enhance the availability of adsorption sites. In all cases, the H–H bond distances remain close to 0.75 Å, consistent with non-dissociative molecular adsorption. These structural characteristics underscore the relevance of using thermodynamically validated GM structures to accurately predict the performance of Si–Li clusters in hydrogen storage.

Table 2 compiles the HOMO–LUMO energy gaps (ΔE_H–L_) of the lithium–silicon clusters studied, both in their bare and hydrogen-adsorbed configurations. These values offer insight into their electronic stability and chemical hardness. Among the bare clusters, Li_4_Si_4_ exhibits the highest gap (3.1 eV), which increases to 3.4 eV upon the adsorption of 8 H_2_ molecules and remains relatively high (3.2 eV) for 12 H_2_ molecules, confirming its closed-shell character and resilience to electronic perturbation. The global minimum structures of Li_6_Si_6_ and Li_10_Si_10_ show gaps of 2.8 and 2.6 eV, respectively, which are also maintained or even slightly enhanced upon hydrogenation, reaching 2.9 eV for both 12H_2_@Li_6_Si_6_ and 30H_2_@Li_10_Si_10_. In contrast, the previously proposed high-symmetry structures—Li_6_Si_6_* (*D_2h_*) and Li_10_Si_10_* (*C_s_*)—exhibit narrower gaps of 2.2 eV and 1.8 eV, respectively, with negligible changes upon hydrogen loading. These values match closely with those reported by Jaiswal et al. [17], who found ΔE_H–L_ values of 2.33 eV for Li_6_Si_6_ and 1.81 eV for Li_10_Si_10_ using the B3LYP/6-31G(d,p) level of theory. Although method-dependent differences are expected, the trend is consistent: higher-energy isomers on the PES tend to display smaller HOMO–LUMO gaps and reduced electronic stability. Additional insights are observed in other systems. Li_8_Si_8_ displays an increase from 2.7 to 3.1 eV as the H_2_ adsorption progresses up to 16 molecules, followed by a decline to 2.1 eV at 24 H_2_, suggesting a saturation threshold for electronic stabilization. Li_12_Si_12_ maintains gaps above 2.6 eV, while Li_12_Si_5_, the most compact and polarizable structure, shows the lowest gaps (1.3–1.7 eV), which increase modestly with hydrogen coverage.

### 2.3. The Hydrogen Adsorption Energetics, Charge Redistribution, and Storage Capacity

**Figure 3:**
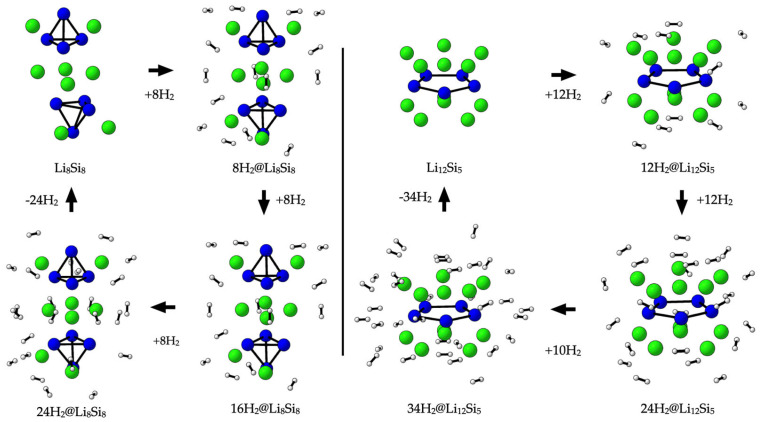
The sequential adsorption of H_2_ molecules over Li_8_Si_8_ and Li_12_Si_5_ clusters.

The hydrogen adsorption properties of the investigated lithium–silicon clusters were assessed through BSSE-corrected adsorption energies (E_ads_), partial charges on Li centers, and gravimetric hydrogen capacities (wt%), as summarized in Table 3. In all systems, Li atoms initially exhibit partial charges between +0.75 and +0.89, consistent with their role as electropositive adsorption sites. Upon hydrogen adsorption, these charges decrease progressively—reaching as low as +0.30 in Li_12_Si_5_—reflecting charge redistribution driven by weak donor–acceptor interactions. The BSSE-corrected E_ads_ values lie within the optimal range for reversible hydrogen storage (−0.11 to −0.16 eV per H_2_), with slightly stronger binding at low coverage. The difference from the uncorrected values (~0.01–0.02 eV) confirms the weakly bound nature of the interactions and the importance of applying BSSE corrections for reliable energetic estimates. All of the PES-validated GM structures—Li_4_Si_4_, Li_6_Si_6_, Li_8_Si_8_, and Li_10_Si_10_—reach 14.72 wt% through adsorbing 12, 18, 24, and 30 H_2_ molecules, respectively. These values align with the upper limit of approximately three H_2_ molecules per Li^+^, as Pan, Merino, and Chattaraj proposed [18]. Li_4_Si_4_, a symmetric and modular unit (*T_d_*), and its oligomeric derivatives Li_8_Si_8_ and Li_12_Si_12_ maintain favorable adsorption behavior and extended capacities, with Li_12_Si_12_ reaching 14.72 wt% with 36 H_2_. While the high-symmetry isomers of Li_6_Si_6_ (*D_2h_*) and Li_10_Si_10_ (*C_s_*) achieve comparable hydrogen uptake and adsorption energetics, they correspond to higher-energy local minima and show narrower Li charge distributions relative to the GMs. The Li_12_Si₅ GM attains the highest capacity in the series (23.45 wt% with 34 H_2_), enabled by its compact *D_5h_* geometry, slightly stronger adsorption energies, and broader Li charge range (+0.30 to +0.78), which reflects increased electrostatic heterogeneity across adsorption sites. For example, Figure 3 illustrates the stepwise hydrogen uptake over Li_8_Si_8_ and Li_12_Si_5_, showing the progressive occupation of Li^+^ centers with minimal structural deformation. These results demonstrate that the Si–Li GMs studied here combine thermodynamic stability, favorable charge redistribution, and optimal adsorption energetics to enable efficient and reversible hydrogen storage.

### 2.4. Thermal Stability and Hydrogen Release Dynamics via BOMD Simulations

**Figure 4:**
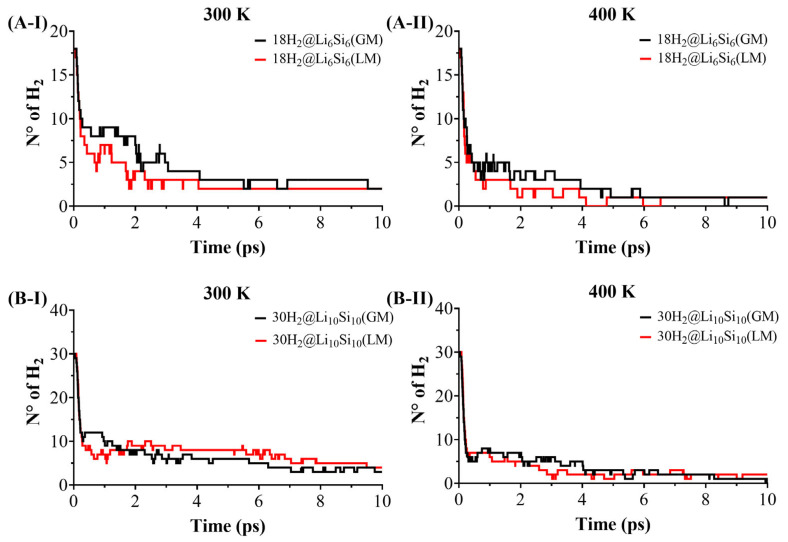
Hydrogen desorption dynamics of 18H_2_@Li_6_Si_6_ and 30H_2_@Li_10_Si_10_ clusters. Snapshots are shown after 10 ps of BOMD simulation at 300 K (**A-I**,**B-I**) and 400 K (**A-II**,**B-II**) for both global minimum (GM) and local minimum (LM) structures. The LM configurations correspond to the geometries reported by Jaiswal et al. [17].

**Figure 5:**
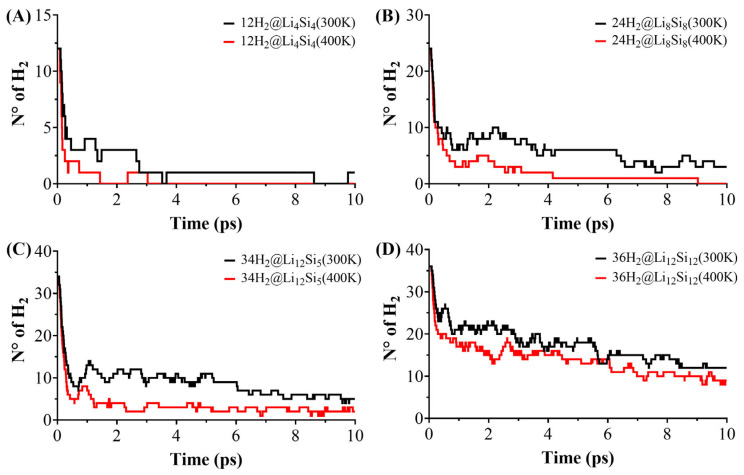
Hydrogen desorption profiles of selected H_2_-loaded Li–Si clusters following 10 ps of BOMD simulation at 300 K and 400 K. Shown are final geometries for (**A**) 12H_2_@Li_4_Si_4_, (**B**) 24H_2_@Li_8_Si_8_, (**C**) 34H_2_@Li_12_Si_5_, and (**D**) 36H_2_@Li_12_Si_12_.

The reversibility and thermal resilience of hydrogen adsorption were assessed through Born–Oppenheimer molecular dynamics (BOMD) simulations on hydrogen-loaded Si–Li clusters: 12H_2_@Li_4_Si_4_, 18H_2_@Li_6_Si_6_, 24H_2_@Li_8_Si_8_, 30H_2_@Li_10_Si_10_, 34H_2_@Li_12_Si_5_, and 36H_2_@Li_12_Si_12_. The simulations were conducted for 10 ps at 300 K and 400 K to probe the hydrogen release under operating conditions. As shown in Figure 4 and Figure 5, desorption behavior depends strongly on cluster size. At 300 K, smaller clusters such as Li_4_Si_4_ and Li_6_Si_6_ release the majority of their adsorbed hydrogen within the first few picoseconds, with only 1–2 H_2_ molecules retained by the end of the simulation. In contrast, larger systems like Li_12_Si_5_ and Li_12_Si_12_ retain 6 and 12 H_2_ molecules, respectively, under identical conditions. This size-dependent stability becomes even more pronounced at 400 K, where the small clusters undergo complete or near-complete desorption, while Li_12_Si_12_ maintains nearly one-third of its original hydrogen load. These observations confirm that increasing the cluster size and coordination density enhances the hydrogen retention under elevated thermal conditions.

A comparative analysis between the PES-validated global minima (GMs) and previously reported local minimum (LM) structures for Li_6_Si_6_ and Li_10_Si_10_ (Figure 4) further underscores the role of structure optimization. While the initial desorption rates are similar for the GM and LM configurations, the GMs consistently retain more hydrogen at later times. For instance, at 400 K, 18H_2_@Li_6_Si_6_(GM) retains ~2 H_2_ molecules, whereas the LM counterpart undergoes full desorption. Similarly, 30H_2_@Li_10_Si_10_(GM) retains ~4 H_2_, while the LM form releases nearly all of its hydrogen content. These differences, though subtle in kinetics, reveal that GM structures offer more resilient binding sites capable of maintaining adsorbed hydrogen under thermal fluctuations. Altogether, the BOMD results support the conclusion that larger, PES-validated Si–Li clusters—particularly Li_10_Si_10_, Li_12_Si_5_, and Li_12_Si_12_—combine structural stability and dynamic retention, making them promising candidates for reversible hydrogen storage under realistic operating conditions.

### 2.5. Visualization of Non-Covalent Interactions via IGMH Analysis

**Figure 6:**
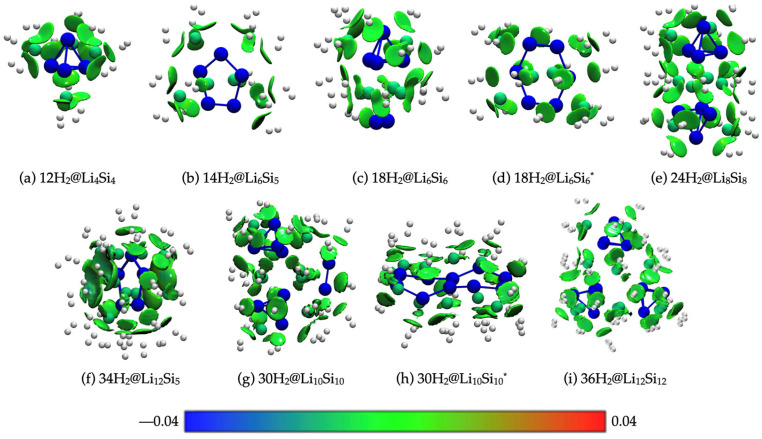
IGMH isosurfaces (δginter = 0.003 a.u.) for hydrogenated Li–Si clusters: (**a**) 12H_2_@Li_4_Si_4_, (**b**) 14H_2_@Li_6_Si_5_, (**c**) 18H_2_@Li_6_Si_6_, (**d**) 18H_2_@Li_6_Si_6_*, (**e**) 24H_2_@Li_8_Si_8_ (**f**) 34H_2_@Li_12_Si_5_, (**g**) 30H_2_@Li_10_Si_10_, (**h**) 30H_2_@Li_10_Si_10_* and (**i**) 36H_2_@Li_12_Si_12_. Calculations were performed at the M06/6-311+G(d,p) level of theory. Blue indicates attractive interactions, green represents weak or dispersive interactions, and red denotes repulsive regions.

To gain visual insight into the nature of the hydrogen adsorption in lithium–silicon clusters, we applied the Independent Gradient Model based on Hirshfeld partitioning (IGMH) to a series of representative hydrogenated systems (Figure 6). As a qualitative method, IGMH enables high-resolution visualization of non-covalent interactions by partitioning the electron density based on the molecular environment, offering superior clarity compared to traditional NCI plots—especially for dispersion-dominated systems. In all cases, the interactions between the H_2_ molecules and Li^+^ centers are characterized by green-colored isosurfaces located between the adsorbates and the cluster surface, confirming that the physisorption is mediated primarily by weak van der Waals forces. No significant blue or red regions are observed, indicating the absence of strong electrostatic attractions or steric repulsion. The interaction fields are more extensive and homogeneously distributed in PES-validated structures such as 12H_2_@Li_4_Si_4_, 24H_2_@Li_8_Si_8_, and 34H_2_@Li_12_Si_5_, reflecting their favorable electrostatic landscapes and high hydrogen uptake. In contrast, the local minima geometries of Li_6_Si_6_ (*D_2h_*) and Li_10_Si_10_ (*C_s_*) exhibit more fragmented interaction regions (18H_2_@Li_6_Si_6_* and 30H_2_@Li_10_Si_10_*), correlating with reduced charge delocalization and lower retention in the BOMD simulations. Overall, the IGMH results reinforce that dispersion-driven, non-dissociative physisorption governs the hydrogen storage in these Si–Li clusters, complementing the energetic and dynamic analyses and validating their potential as reversible hydrogen carriers.

## 3. Computational Details

The potential energy surfaces (PESs) of the Li_6_Si_6_, Li_10_Si_10_, and (Li_4_Si_4_)n (n = 1–3) clusters were explored using the AUTOMATON program [35], which combines a probabilistic automata-based framework with genetic algorithms; for the (Li_4_Si_4_)n oligomers, PES exploration was also carried out using the guided Kick–MEP method [36] inspired by the previous Kick–Fukui method [37]; full methodological details are provided in the Supporting Information. Initial structure screening for all systems was performed for singlet and triplet multiplicities at the PBE0 [38]/SDDALL [39] level. Low-energy isomers within 20.0 kcal·mol^−1^ of the putative global minimum were re-optimized at the PBE0-D3 [40]/def2-TZVP [41] level. Harmonic vibrational frequency calculations confirmed that all of the reported minima were true stationary points. Final relative energies were obtained from single-point refinements at the DLPNO-CCSD(T) [42]/CBS [43]//PBE0-D3/def2-TZVP level using ORCA 5.0.3. All DFT-based geometry optimizations, frequency calculations, and molecular dynamics simulations were performed using Gaussian 16 [44]. Different software packages were selected based on their specific strengths: Gaussian 16 for the structure optimizations, vibrational analysis, and dynamics and ORCA for accurate single-point energies at the post–Hartree–Fock level. Representative low-energy structures are depicted in Figures S1–S3.

Their hydrogen adsorption energetics were evaluated by computing the adsorption energies (E_ads_) for all nH_2_–cluster complexes using the expression

(1) $$E_{ads}=\frac{\left[E\left(complex\right)-{nE(H}_{2})-E(cluster)\right]}{nH_{2}}$$

Here, E(complex) is the total energy of the hydrogen–cluster complex, nE(H_2_) is the energy of n isolated H_2_ molecules, and E(cluster) is the energy of the bare cluster. To improve the accuracy, the adsorption energies were corrected for the basis set superposition error (BSSE) [45] arising from artificial stabilization due to the basis function overlap in weakly bound systems. Boys and Bernardi’s counterpoise (CP) correction method was employed to account for the BSSE [46]. The CP-corrected interaction energy (E_CP_) is given by

(2) $$E_{CP}=E_{Int}-E_{BSSE}$$

Here, E_int_ is the uncorrected interaction energy, and EBSSE is the basis set superposition error correction. All cluster geometries and their hydrogenated analogues were optimized at the M06 [47]/6-311+G(d,p) [48] level, selected for its reliable treatment of the dispersion-driven interactions relevant to hydrogen storage [17]. Practical storage capacity was estimated through gravimetric density calculations at approximate saturation using the expression

(3) $$wt\%=\frac{{M(nH}_{2})}{{M(nH}_{2})+M(cluster)}\times 100$$

Here, M(nH_2_) is the total mass of the adsorbed hydrogen molecules, and M(cluster) is the molecular mass of the bare cluster.

Non-covalent interactions were analyzed using the Independent Gradient Model based on Hirshfeld partitioning (IGMH) [49]. This method improves upon the original IGM method by using Hirshfeld-derived atomic densities, offering a more physically grounded and higher-resolution depiction of weak interactions. Compared to conventional NCI plots, the IGMH provides more detailed insights into dispersion-driven adsorption. All analyses were performed at the M06/6-311+G(d,p) level using Multiwfn 3.8 [50], with visualizations rendered in VMD1.9.4 [51].

To evaluate the thermal resilience and reversibility of hydrogen adsorption, Born–Oppenheimer molecular dynamics (BOMD) [52] simulations were carried out at 300 and 400 K for 10 ps using the Atom-centered Density Matrix Propagation (ADMP) method [53], a Lagrangian-based formulation implemented in Gaussian 16. This approach enables efficient and accurate propagation of the trajectory on the Born–Oppenheimer surface [38].

## 4. Conclusions

This study comprehensively investigates Si–Li clusters as hydrogen storage candidates, emphasizing the importance of accurate potential energy surface (PES) exploration. For the Li_6_Si_6_ and Li_10_Si_10_ systems, global optimization revealed that the true ground-state structures do not correspond to previously proposed benzene- and naphthalene-like motifs but rather consist of compact arrangements built from tetrahedral Si_4_ (*T_d_*) units and a Si_2_ dimer, all stabilized by surrounding lithium atoms. Specifically, Li_6_Si_6_ features a single Si₄ unit and a Si_2_ bridge, while Li_10_Si_10_ includes two Si_4_ units linked via a Si–Si dimer. These findings highlight the necessity of PES validation in cluster-based material design, ensuring that the predicted structures are electronically stable and experimentally feasible.

Guided by the recurrence of the Si_4_–*T_d_* motif, we evaluated oligomeric systems—dimers, trimers, and tetramers—of Li_4_Si_4_ as model cluster-assembled materials (CAMs). These oligomers preserve the local Si_4_ environments and exhibit additive hydrogen storage behavior: each Li_4_Si_4_ unit binds 12 H_2_ molecules (3 per Li^+^), with their total storage capacities increasing proportionally with the number of monomers. Additionally, we explored the aromatic Li_12_Si_5_ sandwich cluster, which incorporates a planar Si_5_ ring stabilized by two Li_6_ caps. This system achieves the highest gravimetric capacity in the series (23.45 wt%), attributable to its high density of accessible Li^+^ sites. Collectively, our results establish clear structure–property relationships among Si–Li clusters and demonstrate the potential of PES-validated, modular architectures for designing thermally stable, high-capacity hydrogen storage materials.

Importantly, this work significantly advances the field of Li–Si clusters for hydrogen storage by delivering (i) a rigorous benchmark of the global minimum structures for key systems previously proposed in the literature, (ii) new modular design principles based on Si₄–*T_d_* building blocks, and (iii) the identification of high-performing sandwich-type and oligomeric clusters with verified thermodynamic and dynamic stability. These contributions provide a robust theoretical foundation for guiding future experimental development of silicon–lithium nanomaterials in next-generation hydrogen storage technologies.

## Figures and Tables

**Table 1:** The computed bond distances (in Å) for Si–Si, Si–Li, Li–H, and H–H interactions in bare Si–Li clusters and their hydrogen-adsorbed complexes, calculated at the M06/6-311+G(d,p) level of theory.

System	$d_{Si-Si}$ (Å)	$d_{Si-Li}$ (Å)	$d_{Li-H}$ (Å)	$d_{H-H}$ (Å)
H_2_	-	-	-	0.74
Li_4_Si_4_	2.44	2.51	-	-
4H_2_@Li_4_Si_4_	2.44	2.51–2.53	2.10	0.75
8H_2_@Li_4_Si_4_	2.44	2.52–2.53	2.12	0.75
12H_2_@Li_4_Si_4_	2.44	2.54–2.55	2.15–2.18	0.75
Li_6_Si_6_ (*)	2.31–2.36	2.40–2.76	-	-
6H_2_@Li_6_Si_6_	2.31–2.35	2.41–2.76	2.09–2.16	0.75
12H_2_@Li_6_Si_6_	2.31–2.34	2.42–2.73	2.08–3.58	0.75
18H_2_@Li_6_Si_6_	2.31–2.34	2.42–2.75	2.09–3.47	0.75
Li_6_Si_6_	2.12–2.49	2.52–2.68	-	-
6H_2_@Li_6_Si_6_	2.12–2.48	2.50–2.69	2.10–2.20	0.75
12H_2_@Li_6_Si_6_	2.12–2.47	2.50–2.71	2.11–2.42	0.75
18H_2_@Li_6_Si_6_	2.12–2.48	2.52–2.73	2.14–3.41	0.75
Li_8_Si_8_	2.35–2.54	2.44–2.89	-	-
8H_2_@Li_8_Si_8_	2.36–2.52	2.47–2.73	2.09–2.26	0.75
16H_2_@Li_8_Si_8_	2.36–2.51	2.50–2.72	2.12–2.29	0.75
24H_2_@Li_8_Si_8_	2.36–2.51	2.49–2.71	2.14–3.46	0.75
Li_10_Si_10_ (*)	2.26–2.47	2.42–3.35	-	-
10H_2_@Li_10_Si_10_	2.26–2.47	2.42–3.23	2.09–2.22	0.75
20H_2_@Li_10_Si_10_	2.27–2.43	2.46–3.14	2.09–3.72	0.75
30H_2_@Li_10_Si_10_	2.27–2.43	2.44–3.21	2.08–3.78	0.75
Li_10_Si_10_	2.13–2.53	2.47–2.85	-	-
10H_2_@Li_10_Si_10_	2.13–2.52	2.46–2.94	2.09–2.25	0.75
20H_2_@Li_10_Si_10_	2.13–2.51	2.49–2.85	2.12–3.55	0.75
30H_2_@Li_10_Si_10_	2.13–2.50	2.51–2.85	2.13–3.90	0.75
Li_12_Si_5_	2.57	2.51–2.56	-	-
12H_2_@Li_12_Si_5_	2.46–2.57	2.49–2.59	1.93–2.17	0.75
22H_2_@Li_12_Si_5_	2.46–2.56	2.50–2.58	1.91–3.65	0.75
24H_2_@Li_12_Si_5_	2.44–2.56	2.50–2.60	2.13–3.81	0.75
32H_2_@Li_12_Si_5_	2.45–2.56	2.50–2.59	1.94–3.76	0.75
34H_2_@Li_12_Si_5_	2.46–2.55	2.50–2.60	2.00–3.50	0.75
Li_12_Si_12_	2.39–2.52	2.47–2.71	-	-
12H_2_@Li_12_Si_12_	2.38–2.48	2.47–2.72	2.10–2.30	0.75
24H_2_@Li_12_Si_12_	2.37–2.52	2.47–2.67	2.11–3.20	0.75
36H_2_@Li_12_Si_12_	2.36–2.52	2.49–2.67	2.13–3.47	0.75

* The local minimum obtained from the study by Jaiswal et al. [17].

**Table 2:** HOMO–LUMO energy gaps (ΔE_H–L_, in eV) for the bare and hydrogen-adsorbed lithium–silicon clusters, calculated at the M06/6-311+G(d,p) level of theory.

System	E_HOMO_	E_LUMO_	ΔE_H-L_
Li_4_Si_4_	−4.4	−1.3	3.1
4H_2_@Li_4_Si_4_	−4.3	−1.0	3.3
8H_2_@Li_4_Si_4_	−4.3	−0.9	3.4
12H_2_@Li_4_Si_4_	−4.2	−1.0	3.2
Li_6_Si_6_ (*)	−3.6	−1.4	2.2
6H_2_@Li_6_Si_6_	−3.6	−1.4	2.2
12H_2_@Li_6_Si_6_	−3.5	−1.2	2.3
18H_2_@Li_6_Si_6_	−3.6	−1.3	2.3
Li_6_Si_6_	−4.6	−1.8	2.8
6H_2_@Li_6_Si_6_	−4.5	−1.6	2.9
12H_2_@Li_6_Si_6_	−4.4	−1.5	2.9
18H_2_@Li_6_Si_6_	−4.6	−1.8	2.8
Li_8_Si_8_	−4.4	−1.7	2.7
8H_2_@Li_8_Si_8_	−4.4	−1.3	3.1
16H_2_@Li_8_Si_8_	−4.3	−1.2	3.1
24H_2_@Li_8_Si_8_	−3.4	−1.3	2.1
Li_10_Si_10_ (*)	−3.3	−1.5	1.8
10H_2_@Li_10_Si_10_	−3.2	−1.4	1.8
20H_2_@Li_10_Si_10_	−3.2	−1.4	1.8
30H_2_@Li_10_Si_10_	−3.2	−1.4	1.8
Li_10_Si_10_	−4.3	−1.7	2.6
10H_2_@Li_10_Si_10_	−4.2	−1.9	2.2
20H_2_@Li_10_Si_10_	−4.1	−1.2	2.9
30H_2_@Li_10_Si_10_	−4.1	−1.2	2.9
Li_12_Si_5_	−3.0	−1.7	1.3
12H_2_@Li_12_Si_5_	−2.8	−1.2	1.6
22H_2_@Li_12_Si_5_	−2.7	−1.1	1.6
24H_2_@Li_12_Si_5_	−2.7	−1.1	1.6
32H_2_@Li_12_Si_5_	−2.8	−1.1	1.7
34H_2_@Li_12_Si_5_	−2.9	−1.2	1.7
Li_12_Si_12_	−4.0	−1.6	2.4
12H_2_@Li_12_Si_12_	−3.9	−1.3	2.6
24H_2_@Li_12_Si_12_	−3.9	−1.3	2.6
36H_2_@Li_12_Si_12_	−3.9	−1.3	2.6

* The local minimum obtained from the study by Jaiswal et al. [17].

**Table 3:** Partial atomic charges on lithium centers (qLi) and adsorption energies without (E_ads_) and with BSSE correction (E_ads+BSSE_) for hydrogen-adsorbed Li–Si clusters, computed at the M06/6-311+G(d,p) level of theory.

System	Q (Li)	$E_{ads+BSSE}$ (eV)	$E_{ads}$ (eV)	wt%
Li_4_Si_4_	0.86	-	-	-
4H_2_@Li_4_Si_4_	0.84	−0.12	−0.12	5.44
8H_2_@Li_4_Si_4_	0.82	−0.12	−0.12	10.32
12H_2_@Li_4_Si_4_	0.81	−0.11	−0.12	14.72
Li_6_Si_6_ (*)	0.83–0.84	-	-	-
6H_2_@Li_6_Si_6_	0.81–0.85	−0.13	−0.14	5.44
12H_2_@Li_6_Si_6_	0.81–0.82	−0.13	−0.13	10.30
18H_2_@Li_6_Si_6_	0.82–0.83	−0.11	−0.11	14.72
Li_6_Si_6_	0.70–0.87	-	-	-
6H_2_@Li_6_Si_6_	0.70–0.84	−0.12	−0.13	5.44
12H_2_@Li_6_Si_6_	0.72–0.82	−0.11	−0.12	10.30
18H_2_@Li_6_Si_6_	0.72–0.81	−0.10	−0.11	14.72
Li_8_Si_8_	0.71–0.88	-	-	-
8H_2_@Li_8_Si_8_	0.72–0.85	−0.12	−0.13	5.44
16H_2_@Li_8_Si_8_	0.73–0.81	−0.11	−0.12	10.32
24H_2_@Li_8_Si_8_	0.77–0.81	−0.10	−0.11	14.72
Li_10_Si_10_ (*)	0.74–0.87	-	-	-
10H_2_@Li_10_Si_10_	0.75–0.85	−0.14	−0.14	5.44
20H_2_@Li_10_Si_10_	0.75–0.83	−0.13	−0.13	10.32
30H_2_@Li_10_Si_10_	0.76–0.83	−0.11	−0.12	14.72
Li_10_Si_10_	0.71–0.89	-	-	-
10H_2_@Li_10_Si_10_	0.72–0.85	−0.12	−0.13	5.44
20H_2_@Li_10_Si_10_	0.73–0.83	−0.11	−0.12	10.32
30H_2_@Li_10_Si_10_	0.74–0.82	−0.10	−0.10	14.72
Li_12_Si_5_	0.30–0.78	-	-	-
12H_2_@Li_12_Si_5_	0.63–0.84	−0.16	−0.17	9.76
22H_2_@Li_12_Si_5_	0.60–0.82	−0.13	−0.14	16.54
24H_2_@Li_12_Si_5_	0.59–0.81	−0.14	−0.14	17.78
32H_2_@Li_12_Si_5_	0.60–0.82	−0.12	−0.13	22.38
34H_2_@Li_12_Si_5_	0.63–0.82	−0.11	−0.12	23.45
Li_12_Si_12_	0.75–0.89			
12H_2_@Li_12_Si_12_	0.76–0.85	−0.11	−0.12	5.44%
24H_2_@Li_12_Si_12_	0.77–0.84	−0.11	−0.12	10.32%
36H_2_@Li_12_Si_12_	0.78–0.83	−0.11	−0.11	14.72%

* The local minimum obtained from the study by Jaiswal et al. [17].

## Data Availability

Data is contained within the article or Supplementary Material.

## Supplementary Materials

The following supporting information can be downloaded at https://www.mdpi.com/article/10.3390/molecules30102163/s1. Figures S1–S3: Putative global minimum and low-lying isomers of Li_6_Si_6_, Li_10_Si_10_, and Li_12_Si_12_. Figure S4: H_2_ adsorption configurations. Table S1: T_1_ diagnostics. Table S2: Basis set superposition error. Table S3: Cartesian coordinates of the optimized Li_4_Si_4_, Li_8_Si_8,_ Li_6_Si_6_, Li_10_Si_10_, and Li_12_Si_12_ structures at the PBE0-D3/def2-TZVP level.
