# Supplementary material for: From Aromatic Motifs to Cluster-Assembled Materials: Silicon–Lithium Nanoclusters for Hydrogen Storage Applications
Source: Molecules. 2025 May 14;30(10):2163. doi: 10.3390/molecules30102163 (PMC12113802; doi:10.3390/molecules30102163)
Supplement: Supplementary file 1 [file molecules-30-02163-s001.zip › molecules-3618567-supplementary.pdf]

Supplementary material

From Aromatic Motifs to Cluster-Assembled Materials: Silicon–Lithium Nanoclusters for Hydrogen Storage Applications

Williams García-Argote [1], Erika Medel [2], Diego Inostroza [3], Alejandro Vásquez-Espinal [4], Luis Leyva-Parra [5], José Solar-Encinas [6], Lina María Ruiz [7,*], Osvaldo Yañez [8] and William Tiznado [1,*]

[1] Centro de Química Teórica & Computacional (CQT&C), Facultad de Ciencias Exactas, Departamento de Ciencias Químicas, Universidad Andrés Bello, Avenida República 275, Santiago 837014, Chile; w.garcaargote@uandresbello.edu (W.G.-A.)
[2] Departamento de Química, División de Ciencias Básicas e Ingeniería, Universidad Autónoma Metropolitana, Iztapalapa, CP 09340 CDMX, México; erikamedel@live.com.mx
[3] Departamento de Física, Facultad de Ciencias, Universidad de Chile, Ñuñoa, Santiago 7800024, Chile; dinostro92@gmail.com
[4] Química y Farmacia, Facultad de Ciencias de la Salud, Universidad Arturo Prat, Casilla 121, Iquique 1100000, Chile; alvasquez@unap.cl
[5] Centro de Investigación en Ingeniería de Materiales (CIIM), Facultad de Ingeniería y Arquitectura, Universidad Central de Chile (UCEN), Santa Isabel 1186, Santiago 8370146, Chile; luis.leyva@ucentral.cl
[6] Laboratory of Theoretical Chemistry, Faculty of Chemistry and Biology, University of Santiago de Chile (USACH), Santiago, Chile; jose.solar@usach.cl
[7] Institute of Biomedical Sciences, Faculty of Health Sciences, Universidad Autónoma de Chile, Santiago, Chile
[8] Centro de Modelación Ambiental y Dinámica de Sistemas (CEMADIS), Facultad de Ingeniería y Negocios, Universidad de Las Américas, Santiago 7500975, Chile; oyanez@udla.cl
* Correspondence: lina.ruiz@uautonoma.cl; wtiznado@unab.cl (W.T.);

Figure S1. PBE0-D3/def2-TZVP geometries of the low-lying energy isomer of Li_6Si_6. Relative energies in kcal·mol^{-1} computed at DLPNO-CCSD(T)/CSB//PBE0-D3/def2-TZVP (**bold**), PBE0-D3/def2-TZVP including zero-point energy (ZPE) corrections levels. Point groups and spectroscopic states are also reported.

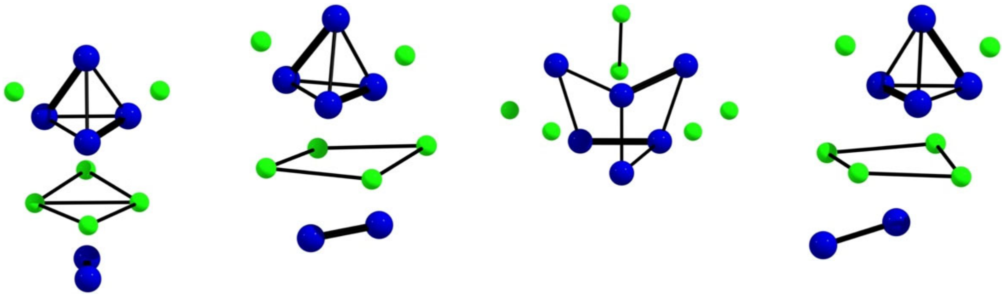

1a (C_s, $^1A'$) **0.0**/1.1 | 1b (C_1, ^1A) **0.7**/2.2 | 1c (C_s, $^1A'$) **3.6**/0.0 | 1d (C_1, ^1A) **3.8**/4.2

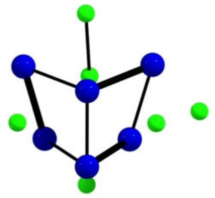

1e (C_1, ^1A)
4.5/1.3

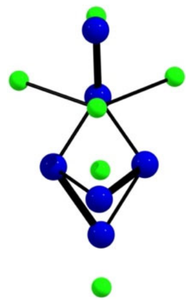

1f (C_s, $^1A'$)
4.8/2.6

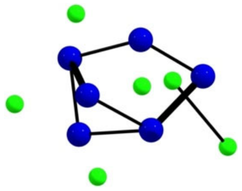

1g (C_1, ^1A)
6.2/3.8

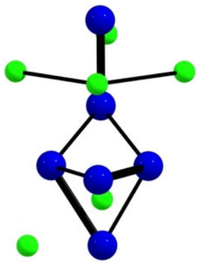

1h (C_s, $^1A'$)
6.5/4.3

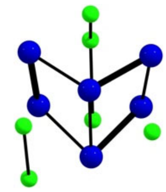

1i (C_1, ^1A)
7.8/3.7

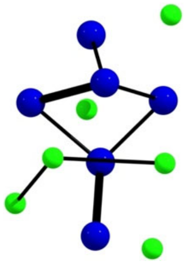

1j (C_1, ^1A)
7.9/6.2

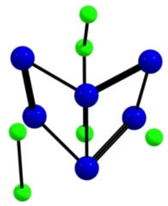

1k (C_1, ^1A)
8.0/3.9

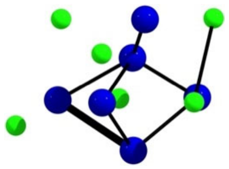

1l (C_1, ^1A)
9.5/5.5

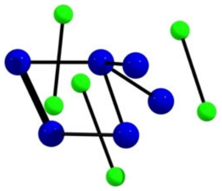

1m (C_1, ^1A)
9.9/6.7

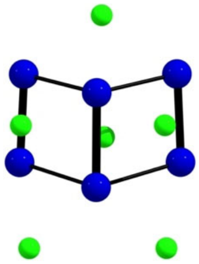

1n (C_s, $^1A'$)
10.3/7.4

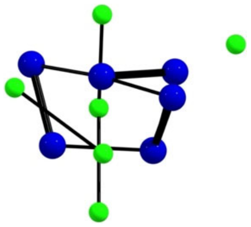

1o (C_1, ^1A)
10.6/7.0

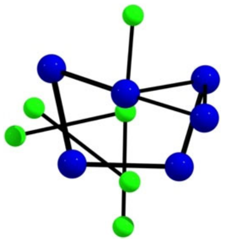

1p (C_1, ^1A)
11.2/6.0

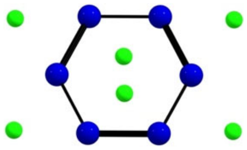

1q (D_{2h}, ^1A_g)
20.4/20.1

Figure S2. PBE0-D3/def2-TZVP geometries of the low-lying energy isomer of $Li_{10}Si_{10}$. Relative energies in kcal·mol^{-1} computed at DLPNO-CCSD(T)/CSB//PBE0-D3/def2-TZVP (**bold**), PBE0-D3/def2-TZVP including zero-point energy (ZPE) corrections levels. Point groups and spectroscopic states are also reported.

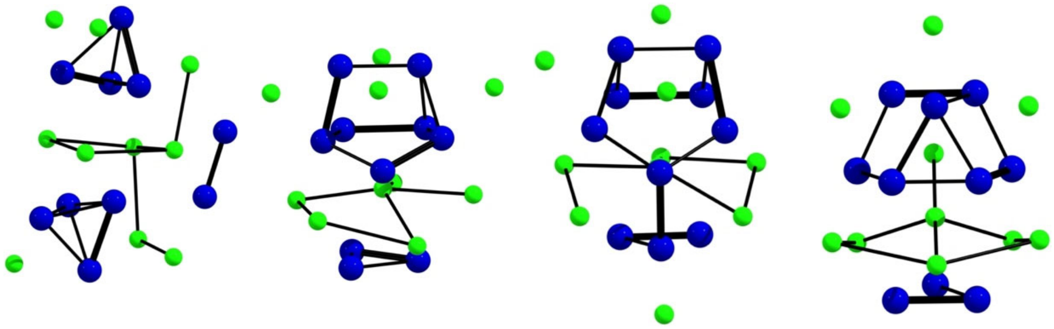

2a (C_1, $^1A'$) **0.0**/0.0 | 2b (C_1, ^1A) **8.9**/1.3 | 2c (C_s, $^1A'$) **9.8**/1.4 | 2d (C_s, ^1A) **10.8**/1.8

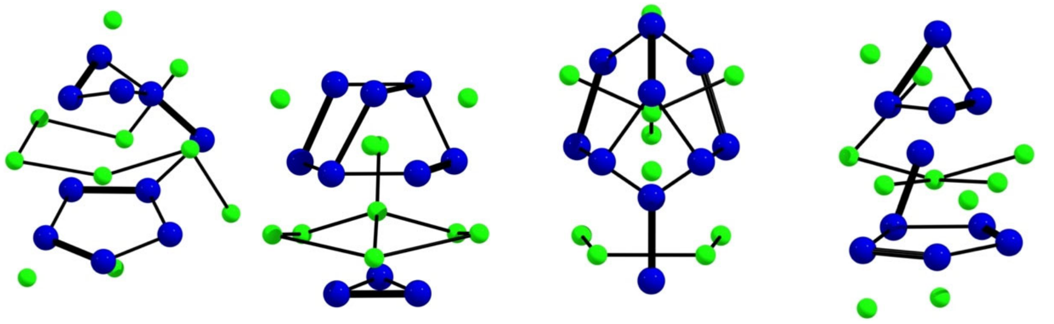

2f (C_1, ^1A) **9.1**/4.0 | 2e (C_1, ^1A) **13.5**/3.7 | 2g (C_1, ^1A) **13.5**/7.0 | 2h (C_s, $^1A'$) **13.5**/7.4

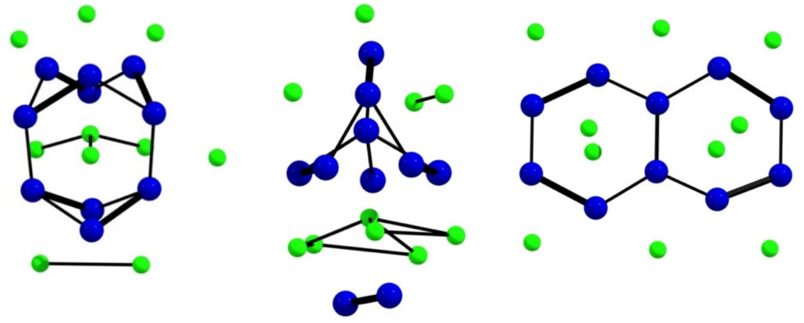

2i (C_1, ^1A) **14.6**/7.6 | 2j (C_1, ^1A) **17.1**/8.2 | 2k (C_s,$^1A'$) **55.3**/52.0

Figure S3. PBE0-D3/def2-TZVP geometries of the low-lying energy isomer of $Li_{12}Si_{12}$. Relative energies in kcal·mol^{-1} computed at DLPNO-CCSD(T)/CSB//PBE0-D3/def2-TZVP (**bold**), PBE0-D3/def2-TZVP including zero-point energy (ZPE) corrections levels. Point groups and spectroscopic states are also reported.

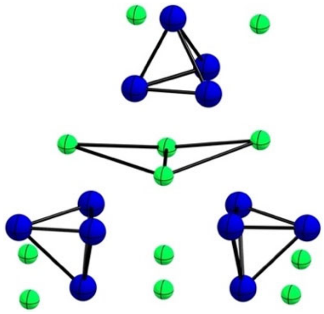

3a (C_1, ^1A)
0.0/0.0

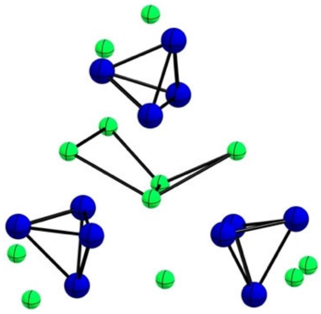

3b (C_1, ^1A)
1.2/2.1

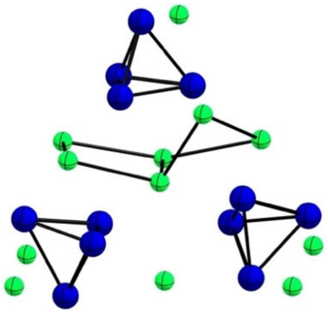

3c (C_1, ^1A)
3.7/2.5

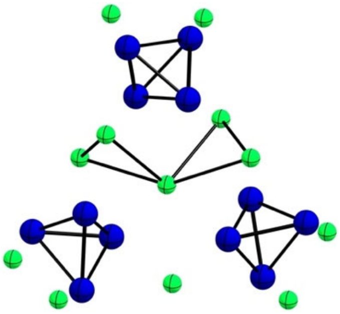

3d (C_2, ^1A)
4.5/7.6

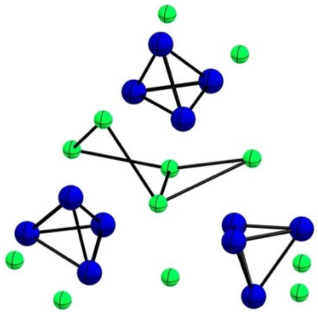

3e (C_2, ^1A)
5.5/5.7

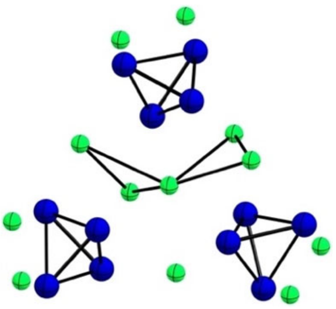

3f (C_1, ^1A)
6.6/**7**.9

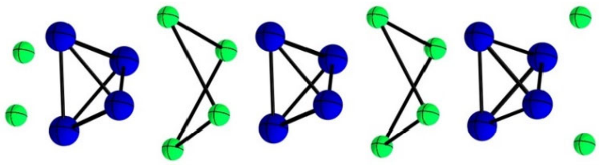

3g (C_{2v}, $^1A_1$)
9.3/8.5

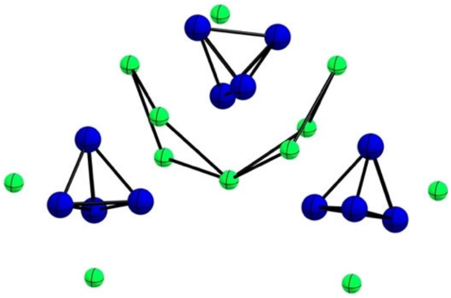

3h (C_1, ^1A)
12.3/10.5

Table S1. Smallest vibrational frequencies in cm^{-1} and T_1 diagnostics for the global minimum of Li_4Si_4, Li_6Si_6, $Li_{12}Si_5$, Li_8Si_8,, $Li_{10}Si_{10}$ and $Li_{12}Si_{12}$.

System	υ_{min}	T_1 Diagnostic
**Li_4Si_4**	95.8	0.01560
**Li_6Si_6(*)**	36.1	0.01645
**Li_6Si_6**	81.4	0.01564
**Li_8Si_8**	39.8	0.01553
**$Li_{10}Si_{10}$(*)**	19.2	0.01896
**$Li_{10}Si_{10}$**	39.3	0.01642
**$Li_{12}Si_5$**	109.7	0.01589
**$Li_{12}Si_{12}$**	25.7	0.01617

Table S2. Minimum frequency (υ_{min}), ZPE and basis set superposition error (E_{BSSE}) of cluster and hydrogenated complexes computed at M06/6-311++G(d,p) level of theory.

System	υ_{min}	ZPE(Hartree)	E_{BSSE}
**Li_4Si_4**	95.8	0.012860	-
**$4H_2@Li_4Si_4$**	37.0	0.064856	0.00083
**$8H_2@Li_4Si_4$**	19.3	0.116660	0.00175
**$12H_2@Li_4Si_4$**	24.1	0.168556	0.00285
**Li_6Si_6(*)**	36.1	0.019048	-
**$6H_2@Li_6Si_6$**	29.0	0.097255	0.001183
**$12H_2@Li_6Si_6$**	38.2	0.177142	0.002155
**$18H_2@Li_6Si_6$**	32.2	0.253593	0.003479
**Li_6Si_6**	81.4	0.019542	-
**$6H_2@Li_6Si_6$**	11.4	0.094756	0.001311
**$12H_2@Li_6Si_6$**	42.5	0.175269	0.002414
**$18H_2@Li_6Si_6$**	20.9	0.251815	0.003944
**Li_8Si_8**	39.8	0.027405	-
**$8H_2@Li_8Si_8$**	37.1	0.131320	0.001723
**$16H_2@Li_8Si_8$**	44.3	0.235574	0.003535
**$24H_2@Li_8Si_8$**	37.4	0.338194	0.005483
**$Li_{10}Si_{10}$(*)**	19.2	0.032473	-

**$10H_2@Li_{10}Si_{10}$**	23.5	0.163685	0.002027
**$20H_2@Li_{10}Si_{10}$**	27.2	0.293433	0.004139
**$30H_2@Li_{10}Si_{10}$**	34.0	0.42212	0.006638
**$Li_{10}Si_{10}$**	39.3	0.033507	-
**$10H_2@Li_{10}Si_{10}$**	18.4	0.161702	0.002228
**$20H_2@Li_{10}Si_{10}$**	18.5	0.292687	0.007474
**$30H_2@Li_{10}Si_{10}$**	31.17	0.421196	0.006642
**$Li_{12}Si_5$**	109.7	0.029119	-
**$12H_2@ Li_{12}Si_5$**	47.2	0.190958	0.003296
**$22H_2@ Li_{12}Si_5$**	33.6	0.319829	0.005310
**$24H_2@ Li_{12}Si_5$**	49.5	0.350761	0.005855
**$32H_2@ Li_{12}Si_5$**	51.1	0.451099	0.007666
**$34H_2@ Li_{12}Si_5$**	25.5	0.474893	0.007879
**$Li_{12}Si_5$**	25.7	0.040677	
**$12H_2@ Li_{12}Si_5$**	20.7	0.195696	0.002981
**$24H_2@ Li_{12}Si_5$**	19.3	0.352116	0.005811
**$36H_2@ Li_{12}Si_5$**	40.1	0.508278	0.008935

* Local minimum obtained from the study of Jaiswal et al.

Figure S4. H_2 adsorption configurations for $nH_2@Li_4Si_4$, $nH_2@Li_6Si_5$, $nH_2@Li_6Si_6$ and $nH_2@Li_8Si_8$ clusters at M06/6-311+G(d,p) level.

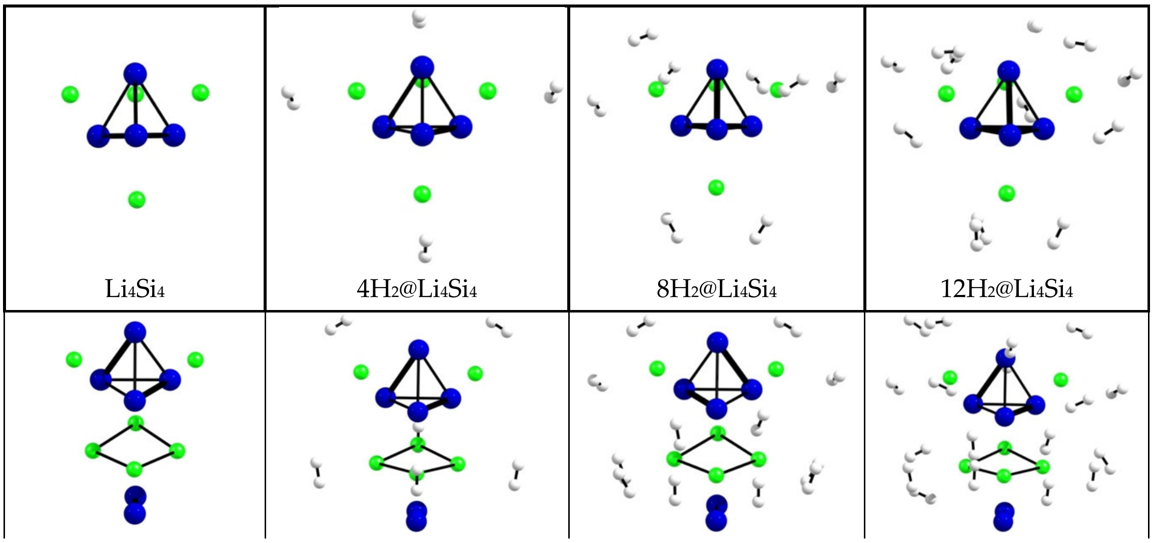

Li_6Si_6	$6H_2@Li_6Si_6$	$12H_2@Li_6Si_6$	$18H_2@Li_6Si_6$
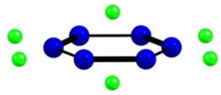	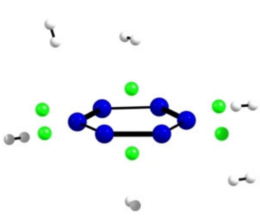	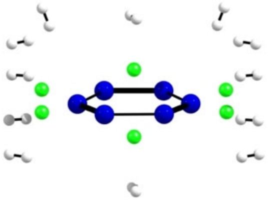	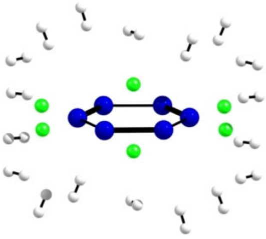
Li_6Si_6(*)	$6H_2@Li_6Si_6^*$	$12H_2@Li_6Si_6^*$	$18H_2@Li_6Si_6^*$
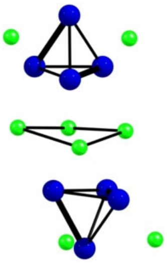	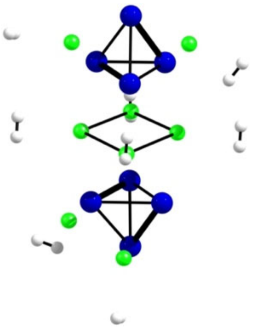	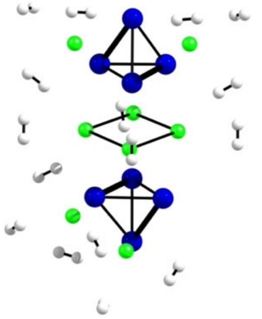	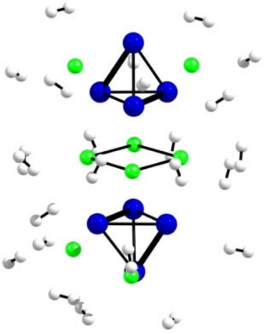
Li_8Si_8	$8H_2@Li_8Si_8$	$16H_2@Li_8Si_8$	$24H_2@Li_8Si_8$
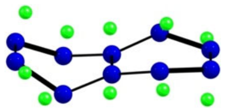	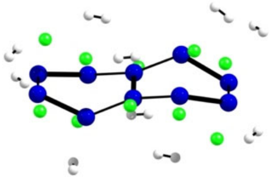	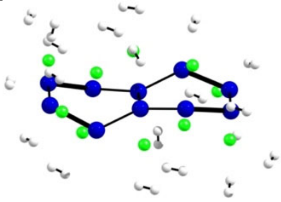	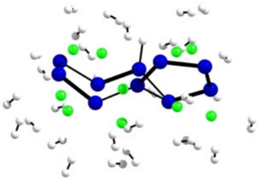
$Li_{10}Si_{10}$(*)	$10H_2@ Li_{10}Si_{10}$	$20H_2@ Li_{10}Si_{10}$	$30H_2@ Li_{10}Si_{10}$
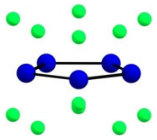	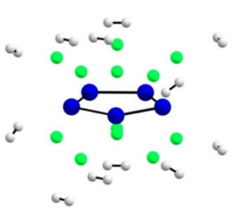	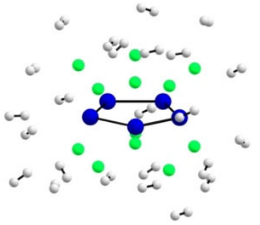	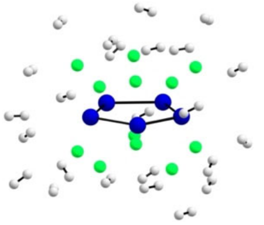
$Li_{12}Si_5$	$12H_2@ Li_{12}Si_5$	$24H_2@ Li_{12}Si_5$	$34H_2@ Li_{12}Si_5$

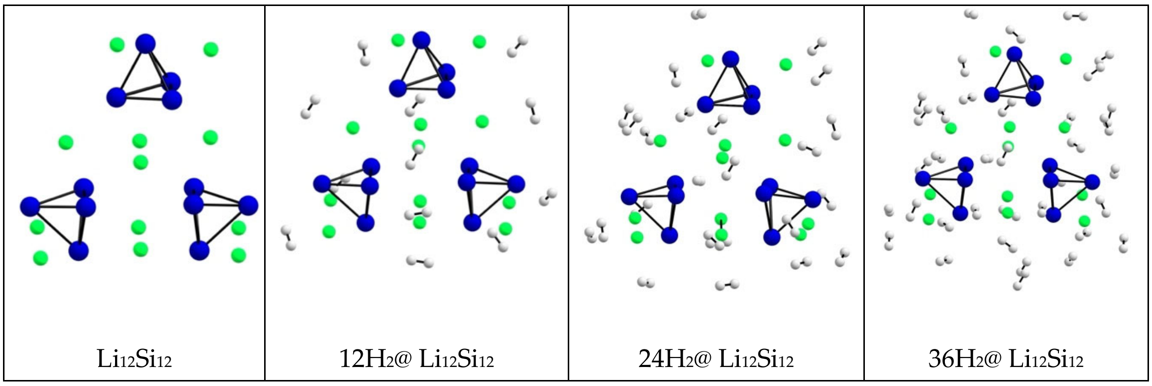

* Local minimum obtained from the study of Jaiswal et al.

Table S3. Cartesian coordinates of H_2-adsorbed complex, (a) $12H_2@Li_4Si_4$, (b) $18H_2@Li_6Si_6$, (c) $18H_2@Li_6Si_6^*$, (d) $24H_2Li_8Si_8$, (e) $30H_2@Li_{10}Si_{10}$, (f) $30H_2@Li_{10}Si_{10}^*$ (g) $34H_2@Li_{12}Si_5$, and (h) $36H_2@Li_{12}Si_{12}$

(a) $12H_2@Li_4Si_4$				(b) $18H_2@Li_6Si_6$			
Li	-1.342623000	2.093677000	-0.806099000	Si	3.418514000	-0.672304000	-0.046813000
Li	1.059465000	0.483721000	2.339178000	Li	-2.379508000	0.330076000	2.028726000
Si	-0.605587000	-0.277854000	-1.338136000	Si	-0.817414000	0.979308000	0.083137000
Si	0.759091000	-1.202795000	0.461358000	Li	1.047981000	2.730189000	0.500767000
Si	0.920116000	1.168373000	-0.101639000	Li	1.345706000	-2.343239000	-0.440643000
Li	-1.627055000	-2.044639000	0.175457000	Si	3.236330000	1.401823000	0.376948000
Si	-1.086519000	0.303617000	0.984606000	Si	-2.721910000	-0.564030000	-0.308956000
Li	1.896375000	-0.545992000	-1.707468000	Li	1.430357000	0.533597000	-1.394549000
H	-0.235256000	3.520088000	-1.980413000	Li	-2.113991000	1.047742000	-2.151557000
H	-0.864694000	3.902211000	-2.145910000	Li	1.293496000	-0.036934000	1.486589000
H	0.804003000	2.148649000	3.913569000	Si	-0.800969000	-1.337193000	0.888957000
H	0.295670000	2.212470000	3.359868000	Si	-0.658933000	-0.913324000	-1.458229000
H	-1.175889000	-3.487934000	1.699106000	H	-3.544178000	-2.467972000	2.445680000
H	-1.836706000	-3.835458000	1.588696000	H	-4.001064000	-2.643131000	3.016501000
H	2.899499000	-2.400492000	-2.577988000	H	-1.578597000	1.130247000	-4.518627000
H	2.475575000	-2.602614000	-1.988815000	H	-1.265290000	0.531743000	-4.181287000
H	-3.685363000	-1.564881000	0.585479000	H	1.228123000	1.867413000	-3.177560000
H	-3.912676000	-2.275005000	0.468604000	H	1.961384000	1.969925000	-3.034123000
H	-2.162204000	3.340573000	0.751425000	H	1.302561000	-0.557021000	3.656962000
H	-2.366519000	3.872597000	0.256471000	H	2.006915000	-0.299464000	3.580622000
H	4.186922000	-0.045601000	-1.765217000	H	0.147348000	4.136500000	1.856746000
H	3.827887000	0.333512000	-1.219962000	H	0.863696000	4.348032000	1.956159000
H	3.229060000	0.861543000	3.057191000	H	1.223944000	-3.411193000	-2.406559000
H	3.179644000	0.821763000	2.305336000	H	1.910472000	-3.677986000	-2.246947000
H	1.159082000	-0.663763000	4.337898000	H	-4.612030000	0.221287000	2.039326000
H	0.852807000	-1.086360000	3.792842000	H	-4.643099000	0.483432000	2.746570000
H	1.584366000	0.111985000	-3.721250000	H	-4.023128000	1.637991000	-3.156099000
H	2.290880000	-0.076715000	-3.907634000	H	-4.187240000	1.103597000	-2.649293000
H	-2.196443000	-3.790671000	-1.237060000	H	2.100486000	-0.757787000	-4.238883000
H	-1.794142000	-3.265912000	-1.601072000	H	1.458324000	-0.904701000	-3.876273000
H	-3.059640000	1.703813000	-2.044256000	H	1.302025000	-4.351504000	0.469187000
H	-3.273760000	2.427132000	-2.016771000	H	2.046122000	-4.235972000	0.457665000
				H	2.977957000	-3.155364000	3.017529000
				H	3.207986000	-2.692819000	2.471921000
				H	1.020515000	4.449120000	-0.925432000
				H	1.741859000	4.234500000	-0.865018000
				H	-3.798716000	2.611553000	0.045239000
				H	-4.332826000	3.143097000	0.045824000
				H	-2.459305000	2.508662000	2.369424000
				H	-2.837761000	2.453517000	3.020656000

				H	-1.588090000	3.217077000	-2.115353000
				H	-1.893463000	3.395918000	-2.782522000
				H	-1.833346000	-0.608919000	3.871059000
				H	-2.259674000	-0.108026000	4.241596000
				H	-0.039823000	1.795021000	3.325922000
				H	0.152740000	2.070369000	3.999664000
				H	-1.414124000	-4.739130000	-1.093092000
				H	-1.391477000	-4.002226000	-0.945790000
(c) $18H_2@Li_6Si_6^*$				(d) $24H_2@Li_8Si_8$			
Si	-2.179109000	-0.202092000	-0.001895000	Li	4.234072000	1.622271000	-0.726657000
Li	3.757046000	-1.671089000	0.040171000	Si	-2.387010000	-0.883495000	1.427796000
Li	-0.003479000	0.034992000	1.342731000	Si	2.216206000	0.302119000	-1.599803000
Si	1.348634000	-1.982722000	0.054271000	Li	-0.700767000	-2.574849000	0.322445000
Li	-3.749562000	1.678857000	-0.030272000	Li	0.510593000	1.942702000	-0.487722000
Li	3.379955000	2.349736000	-0.077310000	Si	-4.183368000	0.105579000	0.211205000
Si	-1.341135000	1.988939000	-0.050893000	Li	-0.248328000	-0.559261000	-1.753212000
Si	2.185830000	0.208352000	0.010623000	Si	-2.604655000	-1.279819000	-0.921555000
Li	-3.371373000	-2.344573000	0.057576000	Si	3.936560000	-0.834136000	-0.398799000
Li	0.010452000	-0.028932000	-1.341229000	Li	0.034443000	0.028388000	1.521173000
Si	0.959870000	2.209025000	-0.050941000	Si	2.418396000	0.751672000	0.820438000
Si	-0.952464000	-2.203115000	0.039044000	Li	3.285326000	-1.321013000	1.978819000
H	-0.120231000	0.295394000	-3.431416000	Si	1.675869000	-1.428136000	-0.046820000
H	0.188578000	-0.392005000	-3.422623000	Si	-1.871577000	0.935747000	-0.149695000
H	-0.231878000	0.354185000	3.425325000	Li	-3.111091000	1.527231000	1.989219000
H	0.243365000	-0.230639000	3.429411000	Li	-3.507366000	0.827436000	-2.094094000
H	-3.300506000	-0.431787000	3.001277000	H	-4.044525000	-0.039419000	-4.208181000
H	-3.570553000	-0.466009000	3.702842000	H	-3.852379000	-0.586471000	-3.724301000
H	-4.438212000	2.742923000	1.642385000	H	5.946182000	2.515933000	0.542167000
H	-5.009130000	2.249683000	1.615390000	H	5.537199000	2.014750000	0.931283000
H	3.513118000	0.451118000	-3.705854000	H	1.840633000	-2.528004000	3.051885000
H	3.252425000	0.421761000	-3.000437000	H	2.298040000	-2.590826000	3.648604000
H	4.608745000	3.342924000	1.381618000	H	-2.651252000	0.957780000	4.023326000
H	3.939190000	3.687402000	1.433558000	H	-2.717037000	1.670442000	4.263194000
H	-3.978503000	-3.641926000	-1.468323000	H	-0.987128000	-3.751931000	2.176957000
H	3.873363000	3.464398000	-1.777399000	H	-0.428275000	-4.200237000	1.940782000
H	-5.108506000	2.292989000	-1.558324000	H	-0.813802000	-0.400908000	-3.952504000
H	-4.533050000	2.777704000	-1.621551000	H	-0.088083000	-0.198677000	-3.994522000
H	4.525093000	3.085938000	-1.734267000	H	-2.053550000	3.467441000	1.866507000
H	-4.621167000	-3.248615000	-1.425335000	H	-2.410808000	3.772345000	2.457585000
H	-3.363358000	-0.746874000	-2.988648000	H	-2.530702000	4.659661000	-1.137207000
H	-3.640105000	-0.923713000	-3.665270000	H	-2.467564000	3.939113000	-0.924068000
H	4.431182000	-2.731603000	-1.639536000	H	4.003688000	-0.366908000	4.108551000
H	5.005591000	-2.242249000	-1.614326000	H	3.769228000	0.148000000	3.607395000
H	4.536140000	-2.773064000	1.631591000	H	5.589131000	1.389800000	-2.395264000

H	5.110449000	-2.286529000	1.572134000	H	5.845813000	2.090203000	-2.287485000
H	-3.874443000	-3.473293000	1.748476000	H	-5.132686000	2.282709000	2.266261000
H	-4.526422000	-3.095447000	1.704964000	H	-4.903564000	2.754568000	2.808942000
H	3.550863000	0.819157000	3.721311000	H	-5.159006000	2.168368000	-2.103320000
H	3.275824000	0.651906000	3.041584000	H	-5.023179000	2.309839000	-2.831867000
H	2.035519000	-2.476406000	-3.037629000	H	-0.049010000	-0.272922000	3.852772000
H	2.259988000	-2.571893000	-3.748923000	H	0.632150000	0.048147000	3.805533000
H	-2.533937000	2.123299000	-3.817137000	H	2.829636000	-2.435672000	-4.161987000
H	-2.265457000	2.085848000	-3.115429000	H	2.785909000	-1.973448000	-3.570459000
H	-2.052188000	2.455773000	3.045616000	H	-1.201551000	-4.390436000	-0.807896000
H	-2.281735000	2.549446000	3.755514000	H	-0.479188000	-4.572714000	-0.694776000
H	2.495711000	-2.239035000	3.822383000	H	0.331709000	4.140901000	0.294856000
H	2.232351000	-2.195129000	3.119118000	H	0.954109000	3.853657000	0.609701000
				H	-2.455096000	2.279226000	-3.778184000
				H	-2.035061000	2.157080000	-3.162747000
				H	0.758418000	3.055391000	-2.480086000
				H	0.222277000	3.551998000	-2.291721000
				H	4.999381000	-2.619204000	2.040930000
				H	4.871115000	-2.750374000	2.772714000
				H	3.410451000	3.637946000	-0.883179000
				H	4.053193000	3.962032000	-1.108036000
				H	0.433097000	-2.885470000	-3.028030000
				H	-0.305679000	-2.960266000	-3.147640000
				H	0.366747000	2.964587000	3.476010000
				H	0.772755000	2.564828000	2.983494000
				H	-3.879346000	-4.523449000	0.947109000
				H	-3.692017000	-3.805425000	0.824817000
				H	3.306219000	3.478413000	2.044524000
				H	3.506855000	4.165454000	2.278912000
(e) $30H_2@Li_{10}Si_{10}^*$				(f) $30H_2@Li_{10}Si_{10}$			
Si	0.088708000	-1.215473000	0.099241000	Li	-4.114171000	-2.890551000	0.644515000
Si	-0.173363000	1.073976000	-0.120579000	Li	4.468350000	-1.285501000	-1.788785000
Si	2.134750000	-2.082863000	0.740879000	Si	-3.854654000	-0.811041000	-0.785530000
Si	4.086210000	-0.747938000	0.220310000	Si	4.077202000	-0.108857000	0.408985000
Si	1.625775000	2.411826000	0.489388000	Si	-2.094097000	-2.480099000	-0.825790000
Si	-2.245992000	1.929895000	-0.632844000	Li	2.574348000	2.314108000	0.443010000
Si	-1.730288000	-2.514985000	-0.489507000	Li	-3.166377000	1.646787000	-1.718268000
Si	3.846051000	1.519289000	0.186624000	Si	2.429686000	0.192804000	-1.327360000
Si	-3.937129000	-1.661846000	-0.004649000	Li	-3.466220000	1.165675000	1.006437000
Si	-4.185941000	0.597691000	-0.007689000	Si	1.749085000	-0.056889000	0.967358000
Li	4.179218000	-3.151844000	-0.289530000	Si	-2.399555000	-1.089162000	1.217025000
Li	-2.062950000	-0.257291000	1.173503000	Li	0.221753000	1.615201000	-1.149494000
Li	-2.407418000	-0.374338000	-1.742787000	Si	-1.577925000	-0.172604000	-0.852901000
Li	-4.410042000	3.033517000	-0.214947000	Si	2.539815000	-1.976503000	-0.241707000

Li	-0.627020000	3.558145000	0.544081000	Li	0.411697000	-1.550012000	-1.893312000
Li	3.267817000	3.676284000	-0.901036000	Li	3.310723000	-1.697577000	2.206757000
Li	0.427357000	-3.778428000	-0.169323000	Si	-1.779297000	3.050792000	0.073203000
Li	-3.377184000	-3.963599000	0.652168000	Si	0.198056000	3.652670000	0.591519000
Li	2.213548000	0.251154000	1.819509000	Li	-0.038244000	-2.004994000	0.816805000
Li	2.028818000	0.126688000	-1.083886000	Li	-0.594556000	1.000669000	1.447733000
H	-4.218474000	5.385381000	-1.220343000	H	-3.799883000	-4.447152000	2.426607000
H	-3.615083000	4.936867000	-1.291266000	H	-3.289989000	-3.889955000	2.429876000
H	-1.228127000	4.177333000	2.534900000	H	-2.249143000	4.430134000	3.928228000
H	-0.474734000	4.129453000	2.578726000	H	-1.947282000	4.293065000	3.253193000
H	4.321322000	5.323592000	0.174216000	H	-2.719631000	2.788242000	-3.488694000
H	4.637904000	4.665930000	0.372151000	H	-2.890164000	2.134611000	-3.823243000
H	5.318580000	-0.214919000	-2.724494000	H	6.212387000	-0.198384000	-2.828628000
H	5.584925000	-0.236922000	-3.428750000	H	5.944872000	0.215217000	-2.256852000
H	0.002646000	-1.816955000	-3.261640000	H	3.782841000	-3.259229000	5.745704000
H	0.286243000	-1.805417000	-3.960502000	H	4.444876000	-3.538588000	5.536872000
H	-4.715906000	-4.819090000	-0.698997000	H	-0.350330000	-3.725388000	2.175117000
H	-4.323866000	-5.459213000	-0.603488000	H	0.381443000	-3.854773000	2.044147000
H	-2.138986000	-0.309010000	3.298109000	H	2.646354000	1.484230000	4.436645000
H	-1.406766000	-0.445905000	3.179120000	H	2.546359000	1.234734000	3.733825000
H	-2.732261000	-0.822925000	-3.802595000	H	0.042828000	2.564906000	-3.212425000
H	-2.691479000	-0.070948000	-3.827356000	H	0.492045000	1.982257000	-3.378809000
H	3.544228000	-3.097230000	-3.005720000	H	0.667085000	-3.630125000	-3.147606000
H	3.125417000	-2.728578000	-2.496981000	H	1.306100000	-3.517036000	-2.763755000
H	2.777185000	0.664585000	3.837566000	H	4.154865000	3.003776000	-0.974551000
H	2.535554000	-0.045848000	3.908088000	H	3.934793000	3.704686000	-0.800834000
H	5.501302000	-3.376827000	1.354581000	H	3.973390000	3.064198000	2.004231000
H	5.400832000	-4.125849000	1.320747000	H	3.471028000	3.626447000	1.994382000
H	3.801523000	3.406452000	-3.122390000	H	4.667506000	-1.390586000	3.981634000
H	4.136679000	2.873410000	-2.702762000	H	4.871529000	-0.907521000	3.438836000
H	-5.508360000	3.397509000	1.526428000	H	4.236391000	-1.690573000	-4.165266000
H	-5.383742000	4.131581000	1.396609000	H	3.655834000	-1.302564000	-3.878330000
H	-4.324510000	-4.379061000	2.473198000	H	-1.798321000	-3.097673000	-3.868574000
H	-3.840157000	-4.957510000	2.495724000	H	-1.574439000	-3.276675000	-4.564156000
H	3.744748000	-2.522933000	3.480042000	H	-5.734399000	1.091808000	0.912113000
H	4.115908000	-2.654768000	4.120046000	H	-5.787154000	1.737909000	1.298906000
H	-0.941407000	4.071058000	-2.482783000	H	-5.913216000	-2.067125000	1.496477000
H	-0.647011000	4.633655000	-2.889835000	H	-6.117466000	-2.739143000	1.771899000
H	6.106678000	-3.499742000	-1.637773000	H	0.792181000	-0.872349000	-4.012736000
H	6.020281000	-2.781053000	-1.417799000	H	0.144582000	-1.214546000	-4.192922000
H	2.090008000	5.308100000	-1.767352000	H	3.616877000	1.866046000	-3.608555000
H	2.710025000	5.611532000	-2.071785000	H	3.916762000	2.255797000	-4.178155000
H	-4.463061000	-1.460008000	3.266071000	H	2.023957000	-1.966559000	4.223596000
H	-4.455628000	-1.571462000	4.010377000	H	1.667813000	-1.500858000	3.746772000
H	-6.083871000	3.553308000	-1.627132000	H	-0.599573000	1.588230000	3.612366000
H	-6.082758000	2.808016000	-1.501821000	H	-0.775878000	0.857050000	3.674068000

H	2.881017000	4.159024000	3.665658000	H	2.066905000	4.650085000	-2.712249000
H	2.639834000	3.807423000	3.045246000	H	1.607726000	4.585031000	-2.120622000
H	4.320100000	-5.314589000	-1.233071000	H	-4.997788000	2.953711000	-1.797302000
H	3.677527000	-5.273979000	-0.842172000	H	-5.300314000	2.290575000	-1.990469000
H	0.952671000	-4.489128000	-2.237573000	H	-5.244272000	-4.723703000	-0.082865000
H	0.230136000	-4.265612000	-2.266520000	H	-4.741988000	-4.518434000	-0.606318000
H	-2.539294000	-3.768413000	-3.175326000	H	3.753007000	-3.829165000	2.885305000
H	-2.739482000	-4.095967000	-3.823398000	H	3.610169000	-3.825417000	2.143681000
H	-1.183449000	-3.153814000	2.780286000	H	3.284533000	6.102892000	0.230687000
H	-1.130847000	-3.339619000	3.508491000	H	2.639402000	5.731135000	0.334704000
H	0.065049000	1.642082000	-4.042947000	H	-5.110227000	4.337647000	1.742139000
H	-0.222268000	1.636241000	-3.346020000	H	-4.475201000	4.191618000	1.366180000
H	2.761640000	0.078035000	-3.040702000	H	-3.633372000	2.285949000	2.963279000
H	2.030413000	0.127564000	-3.224606000	H	-3.830160000	1.595251000	3.192896000
H	0.184137000	1.858757000	3.416095000	H	-1.375870000	-2.327115000	4.742632000
H	-0.072253000	1.822982000	4.123766000	H	-1.566693000	-2.051221000	4.068571000
H	-0.407617000	5.757131000	0.186902000	H	5.865105000	-3.007161000	-2.177063000
H	-1.117343000	5.659279000	-0.044954000	H	5.388351000	-3.206185000	-1.628080000
H	-3.865705000	1.658280000	2.984845000	H	-2.421082000	-0.473315000	-3.862675000
H	-3.769416000	1.923867000	3.683173000	H	-2.424409000	-0.355688000	-4.607262000
(g) $34H_2@Li_{12}Si_5$				(h) $36H_2@Li_{12}Si_{12}$			
Li	-0.639853000	2.885940000	-1.665824000	Li	5.502168000	0.328214000	-0.373499000
Li	2.006184000	-2.137630000	1.584305000	Li	3.874138000	-2.730633000	1.761531000
Li	-2.999328000	0.479706000	-1.722010000	Si	3.288764000	-0.339481000	-1.472596000
Li	-0.166375000	0.080761000	-1.479877000	Si	2.319921000	-2.123575000	-0.227288000
Li	2.422960000	1.089629000	1.960430000	Si	4.667889000	-2.022728000	-0.496840000
Si	0.865406000	1.891394000	0.076481000	Li	0.866470000	-0.538357000	1.413846000
Li	-1.171134000	-2.625893000	1.871139000	Si	3.493933000	-0.366549000	0.998967000
Li	-2.806328000	0.211755000	2.000488000	Li	0.659551000	-0.428452000	-1.661151000
Li	1.827986000	-1.839697000	-2.114727000	Li	-0.038932000	-3.197157000	-0.251523000
Si	0.218880000	-2.119410000	-0.196705000	Li	-4.181275000	-2.372650000	-2.015519000
Si	-1.983808000	-1.054292000	-0.005319000	Si	-1.678536000	-1.681771000	1.137648000
Li	-0.479675000	2.631089000	2.062158000	Si	-3.728861000	-0.968450000	0.065778000
Si	-1.625110000	1.449927000	0.152648000	Si	-1.815457000	-1.576584000	-1.274680000
Li	2.219823000	1.365383000	-2.058371000	Li	-3.973457000	-2.529402000	2.049233000
Li	-1.376752000	-2.363617000	-2.151511000	Si	-2.925826000	-3.338445000	-0.076132000
Li	-0.031185000	-0.135287000	1.437987000	Li	-2.405297000	0.983048000	-1.296477000
Si	2.007655000	-0.281759000	-0.130452000	Li	2.323796000	1.743358000	-0.062361000
H	-0.470432000	-1.129704000	-3.357745000	Li	-1.055798000	4.459965000	-1.807564000
H	-1.202752000	-0.775868000	-3.307250000	Si	0.002636000	2.016353000	1.272632000
H	5.094705000	-0.777577000	-0.707673000	Si	-1.885688000	3.126512000	0.212160000
H	5.830697000	-0.909743000	-0.808435000	Si	0.396694000	4.123690000	0.205479000
H	3.008301000	1.522961000	-4.037094000	Li	-2.321709000	0.839954000	1.505565000
H	2.667886000	2.200259000	-3.978432000	Si	-0.090525000	2.179696000	-1.122751000

H	-0.483724000	5.222335000	-1.349476000
H	0.101737000	4.852829000	-1.046188000
H	-4.814370000	1.395474000	-0.954260000
H	-5.226220000	0.892815000	-1.339092000
H	0.595019000	1.089962000	-3.180562000
H	0.989211000	0.373643000	-3.238547000
H	3.689015000	-3.585066000	1.379411000
H	3.895830000	-2.958847000	1.011954000
H	-0.070675000	-1.519274000	3.076171000
H	-0.794038000	-1.142697000	3.154831000
H	-5.125082000	-0.021941000	1.991042000
H	-4.785391000	-0.554206000	1.575011000
H	-1.431537000	3.189147000	4.050422000
H	-0.794115000	2.835008000	4.247247000
H	3.480555000	1.568304000	3.781177000
H	3.648983000	0.835461000	3.682757000
H	1.409079000	-0.026221000	3.186520000
H	1.025696000	0.687511000	3.273382000
H	-2.085436000	-3.138602000	-4.010824000
H	-1.346088000	-3.309668000	-4.038990000
H	-2.772676000	-4.029090000	1.133855000
H	-2.422356000	-4.631261000	1.424924000
H	-3.246506000	0.309351000	4.116302000
H	-3.622432000	0.941326000	3.939172000
H	4.465586000	1.405939000	0.944514000
H	4.703635000	1.979382000	1.374118000
H	5.101664000	-0.888872000	2.070474000
H	5.753052000	-1.031392000	2.417948000
H	4.373329000	1.945284000	-1.770264000
H	4.038134000	2.602161000	-1.612075000
H	-1.732238000	3.916969000	-3.302916000
H	-1.112088000	3.662508000	-3.651928000
H	-3.598578000	1.010651000	-3.806350000
H	-3.898245000	1.620778000	-3.478115000
H	-1.003498000	4.850129000	2.056423000
H	-1.533032000	4.472442000	1.672426000
H	-1.865243000	-3.293287000	3.750831000
H	-1.210864000	-3.677827000	3.707029000
H	3.411129000	-3.342464000	-2.423812000
H	3.767757000	-2.750544000	-2.121752000
H	-1.460214000	-4.536040000	-1.446640000
H	-2.124462000	-4.680910000	-1.775238000
Li	-0.894464000	4.131139000	2.363693000
H	2.481063000	-3.899692000	3.276789000
H	2.072140000	-3.548449000	2.748366000
H	5.604544000	1.503123000	-2.307884000
H	6.237942000	1.867569000	-2.114856000
H	2.305390000	-3.523268000	-3.794983000
H	2.480159000	-3.151017000	-3.164277000
H	0.203689000	5.070346000	-3.718780000
H	0.562076000	4.559896000	-3.293098000
H	-0.592090000	-3.359353000	3.474749000
H	-0.408298000	-3.715664000	4.112347000
H	-0.793037000	-3.237135000	-3.728773000
H	-0.641234000	-3.602280000	-4.368592000
H	-3.274398000	1.153325000	-3.359899000
H	-3.475402000	1.821056000	-3.076013000
H	-3.456944000	1.065087000	3.434010000
H	-3.432620000	1.771894000	3.173537000
H	-2.970704000	-3.192111000	3.864145000
H	-3.646372000	-3.346605000	4.160422000
H	-3.989765000	-3.379614000	-4.042923000
H	-3.311584000	-3.367385000	-3.712568000
H	0.346423000	5.212932000	3.920034000
H	0.770381000	5.083502000	3.309222000
H	-0.846257000	2.181229000	-4.963977000
H	-0.660402000	2.107610000	-4.238193000
H	3.663918000	3.568001000	2.253934000
H	2.960842000	3.672428000	2.005518000
H	1.527563000	-0.814932000	3.588037000
H	0.821184000	-0.991165000	3.789350000
H	4.902806000	-4.670504000	2.307195000
H	5.105307000	-4.469282000	1.608785000
H	0.470864000	-5.023503000	1.162458000
H	-0.263547000	-4.898314000	1.280740000
H	-5.503272000	-1.101889000	-3.424169000
H	-5.387014000	-0.726035000	-2.780246000
H	-2.166703000	-0.776782000	-4.340235000
H	-2.277458000	-0.605090000	-5.064805000
H	-1.722478000	-0.736327000	4.161358000
H	-1.777255000	-0.512476000	4.878821000
H	-1.426565000	6.710880000	-2.012553000
H	-1.090291000	6.561721000	-1.353782000
H	-2.878536000	4.916166000	2.646243000
H	-2.684539000	5.213600000	3.313233000
H	-5.729226000	-3.897794000	2.509144000
H	-5.440678000	-4.062716000	1.831390000
H	2.664353000	2.573273000	-4.090007000

	H	2.595683000	2.095953000	-3.512330000
	H	5.757728000	1.923081000	1.112134000
	H	6.353141000	2.219343000	0.754282000
	H	7.473225000	-0.513270000	-0.362968000
	H	7.768355000	0.180947000	-0.343542000
	H	-1.050620000	2.649222000	4.178955000
	H	-1.296262000	3.136029000	4.701234000
	H	4.632876000	-2.386720000	4.040951000
	H	4.531176000	-1.742683000	3.658914000
	H	-5.252459000	-0.935796000	2.927881000
	H	-5.336084000	-1.318266000	3.573281000
	H	-2.915468000	4.537694000	-3.037687000
	H	-2.444973000	4.545272000	-3.625095000
	H	-6.167326000	-3.559614000	-2.213457000
	H	-5.810527000	-3.712081000	-1.566091000
	H	-4.783558000	3.019352000	-1.208290000
	H	-5.415513000	2.929579000	-1.606977000
	H	-4.840094000	3.059822000	1.433310000
	H	-5.504903000	3.035893000	1.784302000
	H	3.279145000	3.690709000	-0.861850000
	H	3.803287000	3.186821000	-1.066564000
	H	1.647297000	2.087687000	3.932115000
	H	1.873822000	2.162722000	4.645511000
	H	0.357111000	-0.593110000	-3.928882000
	H	1.107697000	-0.584228000	-3.845458000
	H	0.673356000	-4.715960000	-1.698209000
	H	-0.064876000	-4.871066000	-1.715274000
